# Teachers’ competence, self-efficacy, and their attitudes toward generative AI in education: a correlational study

**DOI:** 10.3389/fpsyg.2026.1756148

**Published:** 2026-05-26

**Authors:** Emmanuel Nana Kwesi Ofori Darko, Zhanyong Qi, Zhiyuan Wang

**Affiliations:** School of Education, Shaanxi Normal University, Xi’an, China

**Keywords:** digital competence, educational technology, generative AI, self-efficacy, teacher attitudes

## Abstract

**Background:**

The rapid integration of Generative Artificial Intelligence (GenAI) offers new opportunities and challenges for education, with teacher adoption being a key factor for successful implementation. However, the psychological factors influencing teachers’ attitudes toward these tools remain poorly understood. This study explores the relationship between K-12 teachers’ digital competence, their self-efficacy for AI integration, and their attitudes toward using GenAI in education.

**Methods:**

A quantitative, cross-sectional survey design was used. Data were collected from 352 K-12 teachers in a large urban school district in Indonesia through an online questionnaire. The survey assessed four constructs: General Digital Competence (GDC), AI-Specific Competence (AISC), Teacher Self-Efficacy for AI Integration (TSE-AI), and Attitudes Toward Generative AI in Education (ATGAI-E). Data analysis involved Pearson’s correlations and hierarchical multiple regression.

**Results:**

The analysis showed strong positive correlations among all variables. Attitudes toward GenAI had the strongest correlation with TSE-AI (*r* = 0.75, *p* < 0.001), followed by AISC (*r* = 0.69, *p* < 0.001) and GDC (*r* = 0.58, *p* < 0.001). The hierarchical regression model explained 62% of the variance in teachers’ attitudes (Adjusted *R*^2^ = 0.61). After accounting for demographic factors, TSE-AI was the most significant predictor (*β* = 0.48, *p* < 0.001), with AISC (*β* = 0.25, *p* < 0.001) and GDC (*β* = 0.11, *p* = 0.015) also contributing.

**Discussion:**

The findings indicate that while both general and AI-specific skills are important, a teacher’s self-efficacy, their confidence in their ability to use GenAI effectively, is the most powerful predictor of their attitude. This suggests that successful GenAI adoption depends more on psychological empowerment than on technical training alone. Professional development programs should therefore focus on building teachers’ confidence through hands-on practice, peer modeling, and supportive feedback to encourage positive engagement with the GenAI classroom.

## 1 Introduction

The educational landscape is undergoing a major shift because of the widespread adoption of Generative Artificial Intelligence (GenAI) (Carruba et al., 2025). Tools like ChatGPT have rapidly moved from niche tools to central topics in public and academic discussions, showing impressive abilities in content creation, summarization, and human-like interaction. In education, GenAI is more than just a small technological addition; it has the potential to disrupt traditional teaching methods, assessment approaches, and key learning principles (Heine and König, 2025). Its uses, from personalized tutoring and real-time content creation to automated feedback and administrative assistance, aim to boost efficiency and offer more customized learning experiences (Mah and Groß, 2024).

The history of educational technology reveals that many innovations fail to achieve widespread and meaningful adoption mainly because of a critical yet often overlooked factor: the teacher (Falebita and Kok, 2025). Teachers, who are the primary decision-makers in the classroom, greatly impact whether new technology is integrated successfully, only used superficially, or completely rejected (Oran, 2023). As school districts develop policies related to GenAI, there remains a significant gap in understanding the factors that influence teachers’ willingness to adopt these tools (Chiu et al., 2025). Although much of the debate focuses on AI’s technical aspects and issues such as academic honesty and bias, less attention has been paid to the psychological factors shaping teachers’ acceptance of these technologies (Falebita and Kok, 2025).

This study addresses the lack of empirical evidence by examining three key areas: teachers’ competence, self-efficacy, and attitudes toward GenAI. According to theories such as the Technology Acceptance Model (TAM), perceived usefulness and ease of use are essential factors influencing the adoption of new technology (Falebita and Kok, 2025; Oran, 2023). These perceptions are influenced by an individual’s skills and confidence levels. Specifically regarding GenAI, this can be broken down into competence and self-efficacy. Teacher competence is complex, encompassing not only General Digital Competence (GDC) but also AI-specific skills (AISC) (Hastomo et al., 2025). The latter includes understanding prompt engineering, critically assessing AI-generated content, and managing related ethical considerations (Hastomo et al., 2025; Oran, 2023).

In addition to having the necessary skills, teacher self-efficacy, the confidence in one’s ability to perform essential tasks, is essential (Chiu et al., 2025). Teacher Self-Efficacy for AI Integration (TSE-AI) refers to a teacher’s belief in their ability to select, adapt, and use GenAI tools to meet instructional goals. Studies consistently indicate that self-efficacy significantly predicts effort, persistence, and success in adopting new classroom technologies (Erol et al., 2025). A teacher may have technical skills but still lack the confidence to integrate GenAI into live lessons. These factors influence a teacher’s overall attitude toward GenAI, which can range from enthusiastic optimism to deep skepticism (Gamlem et al., 2026; Nazim and Alzubi, 2025). Such attitudes are crucial because they affect a teacher’s willingness to learn, experiment with, and incorporate GenAI into their teaching (Xiang et al., 2025).

While it seems logical that competence and self-efficacy positively influence attitudes, the relative impact of these factors remains unclear. Does a teacher’s attitude rely more on their actual skills or their confidence in those skills? Exploring this question has important implications for educational leaders and professional development. If competence is the key factor, training should prioritize technical skills. If self-efficacy is more influential, strategies to enhance confidence should be incorporated into training (Alagöz Hamzaj, 2025; Alvarez et al., 2024). This study employs a quantitative correlational approach to examine the links among K-12 teachers’ general and AI-specific skills, their confidence in integrating AI, and their attitudes toward GenAI in education. The research questions are: (1) How do teachers’ self-rated digital and AI skills relate to their views on GenAI? (2) How does their self-efficacy for AI integration correlate with their attitudes? (3) To what extent do competence and self-efficacy predict attitudes, and which has a stronger influence? Analyzing these relationships aims to provide practical guidance for developing targeted strategies to help teachers adapt to AI’s growing role in education.

## 2 Literature review and conceptual framework

The swift spread of GenAI in education calls for a robust theoretical framework to examine the factors that affect teachers’ adoption. This research combines the Technology Acceptance Model (TAM) and the Technological Pedagogical Content Knowledge (TPACK) framework, with an emphasis on self-efficacy based on Social Cognitive Theory (Mah and Groß, 2024).

### 2.1 Theoretical foundations

The Technology Acceptance Model (TAM) suggests that a person’s willingness to adopt a technology depends on two main beliefs: its usefulness and ease of use. Regarding GenAI, teachers’ perspectives are shaped by their beliefs that these tools can improve their teaching (usefulness) and by their perceptions of how user-friendly they are (ease of use) (Barbieri, 2025). Recent research has often applied and extended TAM to analyze teachers’ adoption of GenAI, frequently adding factors such as self-efficacy, anxiety, and institutional support to develop more detailed models (Li et al., 2026). This study emphasizes competence and self-efficacy as key foundational elements that affect these primary TAM perceptions.

The TPACK framework offers a perspective on the specific knowledge needed for effective technology integration, emphasizing that teachers must combine their knowledge of technology (TK), pedagogy (PK), and content (CK) (Arslankara and Usta, 2024). Effective integration happens at the intersection of these domains. Our study’s constructs, General Digital Competence (GDC) and AI-Specific Competence (AISC), align directly with the TPACK component of Technological Knowledge (TK). A teacher’s skill level in this area is seen as a foundation for developing more advanced knowledge required to use technology effectively for particular subjects. Recent studies have also linked teachers’ TPACK to their readiness to adopt GenAI, indicating that a strong TPACK base correlates with increased willingness and capability to incorporate these innovative tools (Karakaya Özyer and Aydın, 2026).

### 2.2 The central role of self-efficacy

While TAM and TPACK emphasize perceptions and knowledge, Social Cognitive Theory, particularly its self-efficacy component, focuses on beliefs. Self-efficacy refers to an individual’s confidence in their ability to organize and execute actions necessary to achieve a specific goal. This confidence greatly affects motivation, effort, perseverance, and emotional responses to obstacles. In educational technology, teacher self-efficacy is vital for the adoption and effective use of new tools (Alagöz Hamzaj, 2025; Alvarez et al., 2024). Research indicates that teacher self-efficacy for AI Integration (TSE-AI) goes beyond basic skills. A teacher might possess technical abilities (competence) but lack the confidence (self-efficacy) to apply them in the unpredictable classroom environment (Arslankara and Usta, 2024). Recent studies on GenAI highlight the importance of self-efficacy, showing that it directly influences teachers’ willingness to adopt AI, mediates the link between knowledge and behavior, and is a key focus for interventions promoting adoption (Li et al., 2026). Teachers with high self-efficacy are more likely to persist through initial challenges, experiment with new teaching approaches, and see GenAI as a helpful resource rather than a threat (Ahmed et al., 2025). Conversely, low self-efficacy can cause technology anxiety and resistance to change (Li et al., 2025).

### 2.3 Conceptual framework of the study

**Figure 1:**
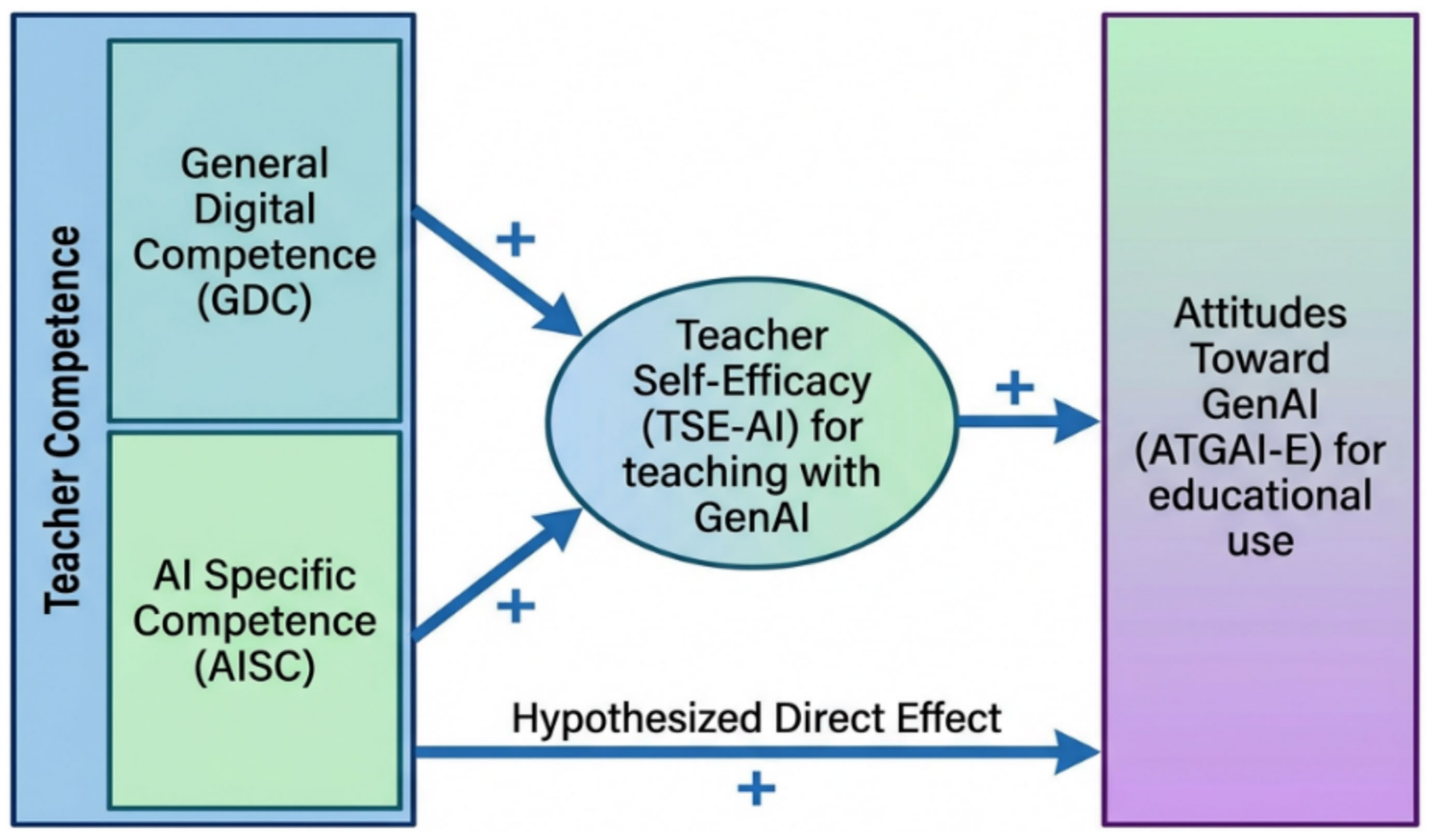
Conceptual framework showing the proposed relationships between teacher competence (GDC, AISC), self-efficacy (TSE-AI), and attitudes toward GenAI (ATGAI-E).

Building on this theoretical synthesis, the study introduces the conceptual framework depicted in Figure 1. It proposes that teachers’ attitudes toward GenAI (ATGAI-E) are linked to their competencies (GDC and AISC) and their self-efficacy (TSE-AI). The framework suggests that all three variables will positively correlate with attitudes, with TSE-AI being the most significant predictor. This highlights that a teacher’s confidence in their abilities plays a crucial role in their readiness to adopt and integrate GenAI.

## 3 Methods

### 3.1 Research design

This study used a quantitative, cross-sectional, correlational research design. This approach is ideal for examining the relationships among multiple variables at a single point in time. The main goal was to assess the strength and direction of associations between the independent variables (General Digital Competence, AI-Specific Competence, and Teacher Self-Efficacy for AI Integration) and the dependent variable (Attitudes Toward Generative AI in Education), as well as the relative predictive power of each variable.

### 3.2 Study setting and participants

Participants were recruited from a large, diverse urban public-school district in Indonesia, the world’s largest archipelago nation, located in Southeast Asia, crossing the equator between the Indian Ocean to the south and west and the Pacific Ocean to the north and east. It comprises over 17,000 islands, including major ones like Sumatra, Java, Borneo (shared with Malaysia and Brunei), Sulawesi, and New Guinea (shared with Papua New Guinea), making it a transcontinental country spanning Asia and Oceania. Geographically, Indonesia is approximately between 6°N and 11°S and 95°E and 141°E, occupying a vast equatorial region that influences its tropical climate and biodiversity. The country’s central point is roughly 2°33′S and 118°01′E, with territory stretching from 6°08′N to 11°15′S and from 94°45′E to 141°05′E. The district serves a broad spectrum of students in elementary, middle, and high schools, representing diverse socioeconomic and cultural backgrounds (Li et al., 2026; Oran, 2023). An invitation to participate in an anonymous online survey was shared via official district channels, including internal newsletters, professional learning community coordinators, and school principals, to ensure broad coverage across K-12. A convenience sampling approach was adopted, resulting in a final sample of 352 K-12 teachers who completed the survey. This sample size aligns with similar studies in educational technology (Arslankara and Usta, 2024).

### 3.3 Instruments

An online questionnaire was developed using Qualtrics, comprising a demographic section and four psychometric scales. All scale items were answered on a 5-point Likert scale from 1 (Strongly Disagree) to 5 (Strongly Agree). The full list of items for each scale is provided in Supplementary Appendix. Digital Competence (GDC): This 10-item scale, adapted from established digital competence frameworks for educators, assesses teachers’ self-rated skills in essential digital tools and pedagogical strategies. A sample item is: “I am confident in my ability to use digital tools to create and share educational resources.” AI-Specific Competence (AISC): Since no standardized instrument was available, the researchers developed this 8-item scale based on emerging AI literacy literature (Erol et al., 2025). It measures teachers’ self-perceived understanding of GenAI concepts, functionalities (e.g., prompt engineering), and ethical considerations. An initial set of items was reviewed by three educational technology experts and pilot-tested with 25 K-12 teachers for clarity and content validity. A sample item is: “I understand the principles of effective prompt engineering to get desired outputs from a generative AI model.” Teacher self-efficacy for AI Integration (TSE-AI): This 10-item scale was adapted from validated measures of teacher self-efficacy in technology integration, with items rephrased to focus on GenAI. It evaluates confidence in using GenAI for instructional purposes. A sample item is: “I am confident I can use generative AI to design engaging learning activities for my students.

While the scale was treated as a unidimensional construct for the primary analysis, consistent with the CFA results, it is theoretically important to decompose teacher self-efficacy for AI Integration (TSE-AI) into several distinct facets. These facets represent the specific domains of confidence required for effective GenAI implementation. Based on the scale items, TSE-AI can be understood through four primary dimensions: (1) Instructional Design Efficacy, or the confidence to plan and integrate GenAI into lessons and curriculum (Items 2, 3); (2) Pedagogical Application Efficacy, which involves using GenAI to support diverse learners and foster higher-order thinking (Items 4, 6, 10); (3) Classroom Management and Assessment Efficacy, referring to the ability to manage student use of GenAI and evaluate their AI-assisted work (Items 5, 7); and (4) Ethical and Technical Efficacy, which covers modeling responsible use and overcoming technical hurdles (Items 1, 8, 9). Attitudes Toward Generative AI in Education (ATGAI-E): This 12-item scale was designed to gage teachers’ overall attitudes toward GenAI, including perceived benefits (e.g., “Generative AI has the potential to personalize learning for students”) and concerns (e.g., “I am concerned that using generative AI will hinder students’ critical thinking skills,” which was reverse-coded).

### 3.4 Psychometric validation and data analysis

Before the main analysis, the psychometric properties of the four scales were evaluated. Internal consistency was measured with Cronbach’s alpha. The unidimensionality of each scale was tested using a one-factor Confirmatory Factor Analysis (CFA) in AMOS 26. As shown in Table 1, all scales exhibited high internal consistency (*α* ≥ 0.89) and acceptable model fit, confirming their appropriateness as single-factor constructs.

**Table 1:** Reliability and CFA model fit indices for measurement scales.

Scale	Items	Cronbach’s *α*	χ^2^	df	CFI	TLI	RMSEA	SRMR
GDC	10	0.92	88.45*	35	0.97	0.96	0.065	0.038
AISC	8	0.94	55.12*	20	0.98	0.97	0.071	0.031
TSE-AI	10	0.95	99.67*	35	0.98	0.97	0.073	0.029
ATGAI-E	12	0.89	112.33*	54	0.96	0.95	0.055	0.045

CFI, Comparative Fit Index; TLI, Tucker-Lewis Index; RMSEA, Root Mean Square Error of Approximation; SRMR, Standardized Root Mean Square Residual. *indicates that the chi-square (χ²) values are statistically significant at *p* < 0.05.

### 3.5 Data analysis

Data analysis was performed using IBM SPSS Statistics 28 through multiple stages. Initially, descriptive statistics for all variables were calculated. Next, Pearson product–moment correlations were used to answer RQ1 and RQ2. A hierarchical multiple linear regression was performed to answer RQ3. Demographic variables (teaching experience, grade level, and subject area) were entered as controls in Block 1, while the primary predictors (GDC, AISC, and TSE-AI) were added in Block 2. This method allows assessment of the unique contribution of psychological constructs beyond teacher background characteristics.

To ensure data quality, procedural and statistical checks were conducted. Participants were assured of anonymity, and the survey scales were separated to reduce common method variance (CMV). Harman’s single-factor test showed that the first unrotated factor accounted for 38.7% of the variance, below the 50% threshold, suggesting that CMV was not a significant concern. Collinearity diagnostics revealed that all tolerance values were above 0.40 and Variance Inflation Factors (VIFs) were below 2.5, confirming the absence of multicollinearity. An alpha level of *p* < 0.05 was used for all significance testing.

## 4 Results

### 4.1 Demographic characteristics of the respondent

Demographic details are shown in Table 2. The sample was predominantly female (73.9%), with a mean age of 41.2 years (SD = 9.8) and an average of 14.5 years of teaching experience (SD = 8.7). Participants were evenly distributed across grade levels and subject areas.

**Table 2:** Demographic characteristics of participants (*N* = 352).

Characteristic	Category	Frequency (N)	Percentage %
Gender	Female	260	73.9
	Male	88	25.0
	Other/prefer not to say	4	1.1
Grade level taught	Elementary (K-5)	124	35.2
	Middle (6–8)	118	33.5
	High (9–12)	110	31.3
Subject area	STEM (Science, Tech, Eng, Math)	105	29.8
	ELA/social sciences	130	36.9
	Arts/humanities/other	117	33.2
Teaching experience	Mean (SD)	14.5 (8.7) years	
Age	Mean (SD)	41.2 (9.8) years	

### 4.2 Descriptive statistics for key variables

Descriptive statistics for the four primary constructs are presented in Table 3. On average, teachers reported the highest level of General Digital Competence (GDC) (*M* = 3.98, SD = 0.65), indicating a solid foundation in general technology use. In contrast, the mean for AI-Specific Competence (AISC) was the lowest (*M* = 3.15, SD = 0.88), suggesting that teachers felt significantly less knowledgeable about the nuances of GenAI. Mean scores for Teacher Self-Efficacy for AI Integration (TSE-AI) (*M* = 3.45, SD = 0.91) and Attitudes Toward GenAI (ATGAI-E) (*M* = 3.51, SD = 0.74) were moderate, hovering slightly above the neutral midpoint of the 5-point scale.

**Table 3:** Descriptive statistics for key variables (*N* = 352).

Variable	M	SD	Min	Max
GDC	3.98	0.65	2.10	5.00
AISC	3.15	0.88	1.00	5.00
TSE-AI	3.45	0.91	1.20	5.00
ATGAI-E	3.51	0.74	1.58	5.00

All variables were measured on a 5-point scale (1 = Strongly Disagree, 5 = Strongly Agree). A higher score indicates a higher level of the construct.

### 4.3 Correlational analysis

To address RQ1 and RQ2, Pearson correlation coefficients were computed to examine the relationships among the variables. As shown in Table 4, all correlations were statistically significant and positive. Both GDC [*r* = 0.58, 95% CI (0.50, 0.65), *p* < 0.001] and AISC [*r* = 0.69, 95% CI (0.62, 0.75), *p* < 0.001] were strongly and positively associated with Attitudes Toward GenAI (ATGAI-E). This indicates that teachers with higher levels of both general and AI-specific competence tend to have more favorable attitudes. TSE-AI had the strongest correlation with ATGAI-E [*r* = 0.75, 95% CI (0.69, 0.80), *p* < 0.001]. This very strong positive relationship suggests that teachers’ confidence in their ability to use GenAI is a key predictor of their overall attitude.

**Table 4:** Pearson correlation matrix for competence, self-efficacy, and attitudes.

Variable	1. GDC	2. AISC	3. TSE-AI	4. ATGAI-E
1. GDC	–			
2. AISC	0.62**	–		
3. TSE-AI	0.65**	0.71**	–	
4. ATGAI-E	0.58**	0.69**	0.75**	–

**p < 0.001. GDC, General Digital Competence; AISC, AI-Specific Competence; TSE-AI, Teacher Self-Efficacy for AI Integration; ATGAI-E, Attitudes Toward GenAI in Education.

### 4.4 Hierarchical regression analysis

**Figure 2:**
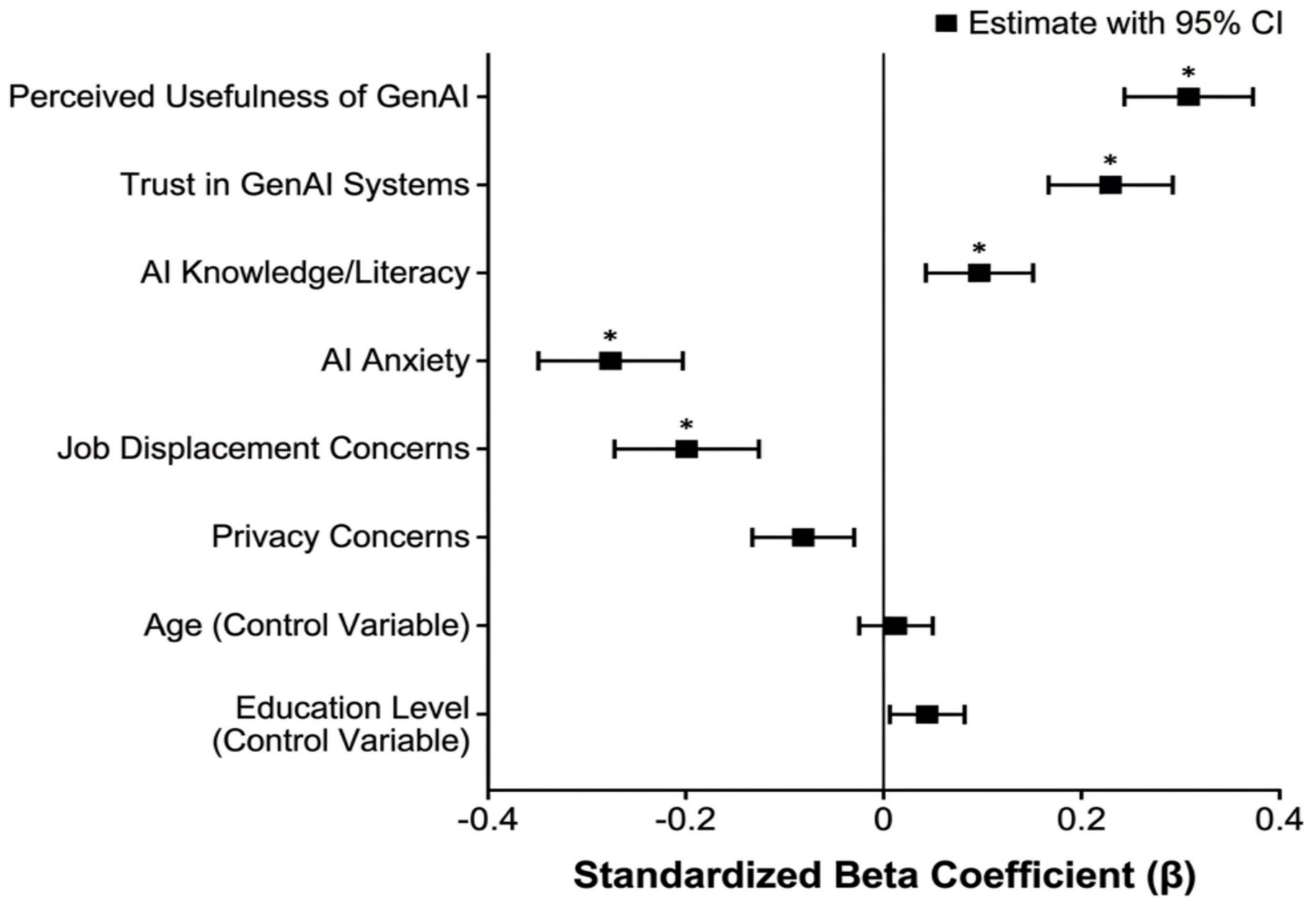
Standardized beta coefficients (*β*) from a regression model predicting attitudes toward Generative AI. Positive values indicate more favorable attitudes; error bars show 95% confidence intervals; *signifies *p* < 0.05, indicating significance. Attitudes toward GenAI are primarily influenced by perceptions (usefulness + trust), knowledge, and fears (anxiety + job loss) rather than demographics. The most effective way to improve acceptance is to demonstrate real usefulness, build trust, reduce anxiety, and address concerns about jobs and privacy. Age and education become less relevant once these psychological factors are taken into account.

**Figure 3:**
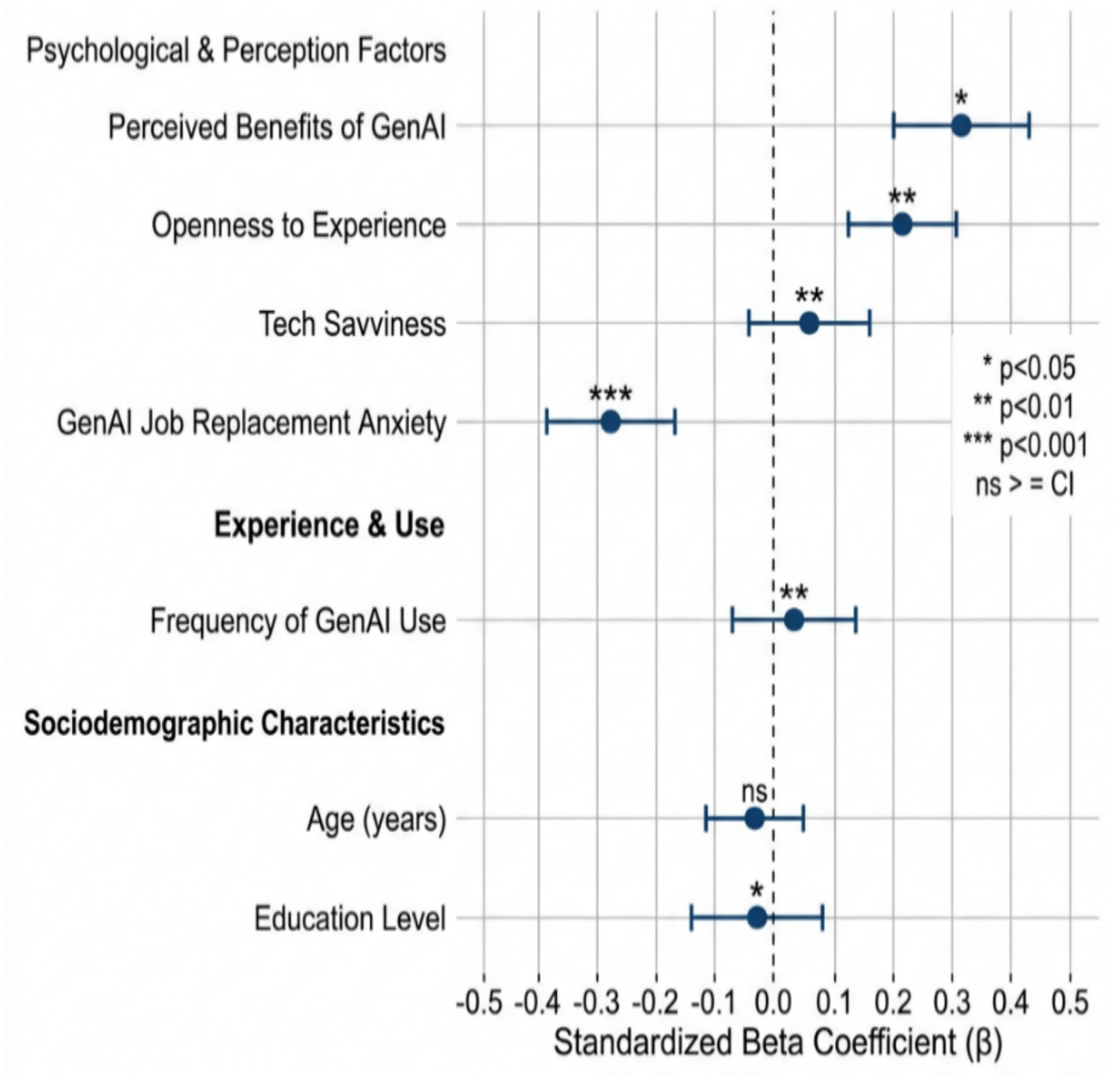
Standardized beta coefficients (β) from the final multiple regression model predicting attitudes toward Generative AI (GenAI). Positive values indicate more favorable attitudes. Error bars represent 95% confidence intervals, with significance marked by asterisks (*p < 0.05, **p < 0.01, ***p < 0.001; ns = interval crosses zero). Attitudes toward GenAI are influenced far more by perceptions, personality, and hands-on experience than by demographics. The model suggests that emphasizing benefits, alleviating job-loss worries, and promoting regular use may be the most effective ways to improve public acceptance. Age, surprisingly, has little to no effect once other factors are accounted for.

To address RQ3, a hierarchical multiple regression analysis was performed to predict teachers’ attitudes (ATGAI-E). The results, summarized in Table 5, showed that in the first step, demographic control variables (teaching experience, grade level, subject area) were included. This model was statistically significant, *F* (3, 348) = 3.15, *p* = 0.025, but only explained a small portion of the variance in attitudes (*R*^2^ = 0.026). In the second step, the three main predictors GDC, AISC, and TSE-AI were added, significantly increasing the explained variance, ΔR^2^ = 0.61, F Change (3, 345) = 185.43, *p* < 0.001. The final model was highly significant, *F* (6, 345) = 94.81, *p* < 0.001, and explained 62% of the total variance in teachers’ attitudes (Adjusted *R*^2^ = 0.61). All three psychological predictors were significant. Notably, TSE-AI emerged as the strongest predictor, with the highest standardized beta coefficient (*β* = 0.48, *p* < 0.001), surpassing AISC (*β* = 0.25, *p* < 0.001) and GDC (*β* = 0.11, *p* = 0.015). This indicates that, after controlling for demographics and other competence factors, a teacher’s self-efficacy is the most important unique predictor of their attitude toward GenAI (Figures 2, 3).

**Table 5:** Hierarchical multiple regression analysis predicting attitudes toward GenAI (ATGAI-E).

Variable	Model 1 (β)	Model 2 (β)
Block 1: controls		
Teaching experience	0.08	0.02
Grade level	−0.11*	−0.04
Subject area	0.05	0.01
Block 2: predictors		
GDC		0.11*
AISC		0.25***
TSE-AI		0.48***
Model summary		
*R* ^2^	0.026	0.639
Adjusted *R*^2^	0.018	0.613
Δ*R*^2^	0.026	0.613
*F*	3.15*	94.81***
ΔF	3.15*	185.43***

*p < 0.05, **p < 0.001. ***means the result is highly significant with *p* < 0.001, indicating very strong statistical evidence for the relationship.

## 5 Discussion

This study aimed to explore the links between K-12 teachers’ competence, self-efficacy, and attitudes toward integrating GenAI. The findings reveal a clear narrative: while technological knowledge is important, a teacher’s confidence in applying that knowledge is the strongest factor influencing their attitude toward GenAI. These insights are valuable for theory, practice, and future research in AI education. The positive correlations between general and AI-specific competence and attitudes support existing technology adoption models, such as TPACK, which emphasize the importance of technological knowledge for successful integration. Naturally, teachers who feel more competent are better at imagining real classroom applications and recognizing benefits, leading to more positive perceptions, which align with studies by Ahmed et al. (2025) and Tomczyk and Majkut (2025). The stronger link with AISC compared to GDC reflects the unique challenges of GenAI: while basic tech skills are helpful, understanding AI’s capabilities and limitations has a greater impact on fostering a positive outlook (Pan and Li, 2025). The strong positive link between self-efficacy and attitudes aligns with extensive research based on Social Cognitive Theory. The correlation (*r* = 0.75) is notably high, indicating a strong association between teachers’ confidence and their perceptions of GenAI. This is supported by recent studies that highlight self-efficacy as key in teachers’ willingness to adopt GenAI (Shi, 2025). Teachers with high self-efficacy tend to view technological challenges as opportunities for growth, creating a positive cycle where confidence drives experimentation, leading to mastery experiences that reinforce efficacy (Hazzan-Bishara et al., 2025).

The most important insight, however, came from hierarchical regression analysis. After accounting for demographic factors, self-efficacy emerged as the strongest predictor of teachers’ attitudes toward GenAI. The standardized beta for TSE-AI (*β* = 0.48) was nearly twice that of AISC (*β* = 0.25) and more than four times that of GDC (*β* = 0.11). This clearly shows that a teacher’s belief in their abilities is the main psychological factor influencing their attitude. This finding extends research that views self-efficacy as a vital link between knowledge and action (Mai and Khue, 2025). It suggests that two teachers with the same skills might have very different attitudes toward GenAI, with the more confident teacher being more likely to see it positively. This underscores that successful GenAI adoption depends as much on psychological empowerment as on technical knowledge (Almusharraf et al., 2025). Lack of confidence can be a major barrier, preventing teachers from engaging deeply enough with GenAI to see its potential, regardless of their actual skills. On the other hand, a strong sense of efficacy can motivate teachers to learn, explore, and develop a constructive and positive outlook (Mai and Khue, 2025).

### 5.1 Deconstructing teacher self-efficacy for AI integration

While the finding that overall self-efficacy is the strongest predictor of attitudes is significant, a more detailed understanding of this concept offers greater practical benefits. By breaking down TSE-AI into the specific areas identified in this study, Instructional Design, Pedagogical Application, Classroom Management, Assessment, and Ethical and Technical Efficacy, we can move from a broad call for “confidence-building” to targeted interventions. For example, a teacher might feel very confident in their ability to design a lesson using a GenAI tool (Instructional Design Efficacy) but feel anxious about assessing student work created with AI help (Assessment Efficacy) or leading discussions on AI ethics (Ethical Efficacy). This layered perspective explains why some teachers who appear technically capable may still hesitate to fully adopt GenAI. Their hesitation might not come from a general lack of confidence but rather from low self-efficacy in specific, high-stakes areas of their practice. Therefore, the main contribution of this study is not only to confirm that self-efficacy is important but also to demonstrate that TSE-AI is a complex, multidimensional belief system. This view shifts the challenge for professional development: the goal is not merely to improve a single score but to identify and strengthen specific areas of low confidence, fostering a stronger, more adaptable sense of efficacy across all aspects of AI integration. This study expands on literature highlighting self-efficacy as vital for technology adoption in education. Its unique focus is breaking down this broad concept into specific, actionable parts. This aligns with frameworks like TPACK, which argue effective technology use combines related skills. Our TSE-AI model complements TPACK by emphasizing teachers’ beliefs about their ability to apply knowledge in an AI-driven classroom. This view explains why some tech-savvy teachers hesitate to adopt AI, not because of ability but because of low confidence in areas like academic honesty and ethical issues. These challenges are major obstacles, highlighting the need for tailored professional development. Instead of generic workshops, schools should focus on specific skills, such as using AI for personalized learning or feedback. Research shows institutional support, including customized training, is vital for fostering confidence and encouraging AI adoption, helping teachers become proficient and adaptable.

### 5.2 Implications for practice and professional development

The study’s findings have practical implications for school leaders and PD planners. Since self-efficacy is vital, PD programs should do more than basic workshops that just demonstrate features. Effective PD must intentionally foster teacher confidence through evidence-based methods: offer hands-on, low-pressure opportunities for teachers to practice using GenAI to address real teaching challenges, such as lesson adjustments or the creation of assessment rubrics. Success in these tasks significantly enhances self-efficacy. Highlight relatable teacher-leaders successfully using GenAI in their classrooms. Seeing peers succeed can inspire the belief, “If they can do it, so can I.” A supportive environment where instructional leaders and colleagues provide specific, constructive feedback and encouragement boosts motivation and resilience. The lower average scores for AISC, combined with the importance of self-efficacy, suggest teacher anxiety is a major barrier. School leaders should foster a culture that minimizes stigma around uncertainty and promotes experimentation. While self-efficacy is key, competence remains crucial, making targeted AI literacy training essential to cover prompt engineering, data privacy, algorithmic bias, and the risks of misinformation.

### 5.3 Limitations and directions for future research

This study has several limitations that indicate directions for future research. First, its cross-sectional design only captures a single moment and cannot establish causality. Long-term, longitudinal studies are needed to track how competence, self-efficacy, and attitudes develop over time. Second, using a convenience sample from one district limits the ability to generalize the findings. Future research should attempt to replicate these results with more diverse samples from different areas. Third, reliance on self-report measures makes the study susceptible to social desirability bias. Future work could incorporate objective, performance-based assessments of competence or observe classroom practices directly. Lastly, as the field of GenAI advances quickly, ongoing research is crucial to keep up with technological changes and their impact on teachers’ perceptions. While this quantitative study identifies key predictive relationships, qualitative research is necessary to deepen the understanding of teachers’ experiences with integrating GenAI.

## 6 Conclusion

Integrating Generative AI into education is inevitable, and its effectiveness depends on teachers’ preparedness and receptivity. Evidence shows that while technical skills matter, teachers’ confidence in their ability to use these tools is the key factor shaping their attitude. Teachers who feel confident are more likely to be open, optimistic, and eager to collaborate with GenAI to enhance teaching and learning. Educational leaders should understand that promoting positive adoption requires investing in empowering teachers. This transition involves moving beyond basic tech training to comprehensive professional development that builds confidence, encourages experimentation in supportive environments, and addresses concerns about transformative change. Strengthening teacher self-efficacy in all areas not only teaches them to operate new tools but also equips them with resilience, creativity, and a positive outlook to help shape the future of education in the AI era. This paper advances the field by integrating research on teacher self-efficacy and GenAI adoption in education. It emphasizes the role of self-efficacy in the adoption of new technologies. Theoretically, it broadens self-efficacy theory within technology acceptance models, highlighting its role in explaining the adoption of innovative technologies. Practically, it guides policymakers and educators to shift from a tool-focused approach to a human-centered strategy that boosts teachers’ confidence, critical thinking, and Pedagogical flexibility for better AI integration.

## Data Availability

The original contributions presented in the study are included in the article/Supplementary material, further inquiries can be directed to the corresponding author.

## References

[ref1] Ahmed S. Faisal M. Hasan S. S. F. Ghazi M. A. (2025). Impact of artificial intelligence (AI) on teacher self-efficacy: a systematic literature review. Soc. Sci. Rev. Archives 3, 3406–3415. doi: 10.70670/sra.v3i4.626

[ref2] Alagöz Hamzaj Y. (2025). Generative AI acceptance among future educators: personality and behavioral insights. Educ. Inf. Technol. 30, 23165–23188. doi: 10.1007/s10639-025-13678-3

[ref3] Almusharraf A. Bailey D. Almusharraf N. Alotaibi T. (2025). Students’ perceptions of generative AI in EFL writing: strategies, self-efficacy, satisfaction and behavioural intention. Australas. J. Educ. Technol. 41, 18–36. doi: 10.14742/ajet.10045

[ref4] Alvarez L. Ortoleva G. Sutter Widmer D. Fritz M. Bugmann J. Boéchat-Heer S. . (2024). Future teachers’ beliefs about generative AI. Assessing technology acceptance as students or as aspiring professionals. J. Technol. Teach. Educ. 32, 383–408. doi: 10.70725/379206cljimb

[ref5] Arslankara V. B. Usta E. (2024). Generative artificial intelligence as a lifelong learning self efficacy: usage and competence scale. J. Teach. Educ. Lifelong Learn. 6, 288–302. doi: 10.51535/tell.1489304

[ref6] Barbieri W. (2025). Generative AI as a “placement buddy”: supporting pre-service teachers in work-integrated learning, self-management and crisis resolution. Australas. J. Educ. Technol. 41, 34–49. doi: 10.14742/ajet.10035

[ref7] Carruba M. C. Caiazzo A. Scuotto C. Savioni L. Triberti S. (2025). A grade for artificial intelligence: a study on school teachers’ ability to identify assignments written by generative artificial intelligence. Cyberpsychol. Behav. Soc. Netw. 28, 489–496. doi: 10.1089/cyber.2024.0524, 40471066

[ref8] Chiu T. K. F. Ahmad Z. Çoban M. (2025). Development and validation of teacher artificial intelligence (AI) competence self-efficacy (TAICS) scale. Educ. Inf. Technol. 30, 6667–6685. doi: 10.1007/s10639-024-13094-z

[ref9] Erol M. Canbeldek Erol M. Erol A. Gök Çolak F. (2025). Exploring the relationship between teachers’ AI attitudes, AI self-efficacy, and AI technological pedagogical content knowledge. Eur. J. Educ. 60:e70332. doi: 10.1111/ejed.70332

[ref10] Falebita O. S. Kok P. J. (2025). Artificial intelligence tools usage: a structural equation modeling of undergraduates’ technological readiness, self-efficacy and attitudes. J. STEM Educ. Res. 8, 257–282. doi: 10.1007/s41979-024-00132-1

[ref11] Gamlem S. M. McGrane J. Brandmo C. Moltudal S. Sun S. Z. Hopfenbeck T. N. (2026). Exploring pre-service teachers’ attitudes and experiences with generative AI: a mixed methods study in Norwegian teacher education. Educ. Psychol. 46, 27–51. doi: 10.1080/01443410.2025.2528663

[ref12] Hastomo T. Widiati U. Ivone F. M. Zen E. L. (2025). Efl teachers’ self-efficacy in using AI tools: a COMPARATIVE study in Indonesia. Adv. Educ. 19, 103–123. doi: 10.20535/2410-8286.335502

[ref13] Hazzan-Bishara A. Kol O. Levy S. (2025). The factors affecting teachers’ adoption of AI technologies: a unified model of external and internal determinants. Educ. Inf. Technol. 30, 15043–15069. doi: 10.1007/s10639-025-13393-z

[ref14] Heine S. König J. (2025). Applying artificial intelligence in teacher education: preservice teachers’ attitudes and reflections in using ChatGPT for teaching and learning. Eur. J. Teach. Educ. 48, 934–963. doi: 10.1080/02619768.2025.2540791

[ref15] Karakaya Özyer K. Aydın B. (2026). Generative artificial intelligence (GenAI) meets assessment: experimental insights into teacher candidates’ attitudes and acceptance. Int. J. Educ. Math. Sci. Technol. 14, 21–45. doi: 10.46328/ijemst.5190

[ref16] Li S. Liu J. Dong Q. (2025). Generative artificial intelligence-supported programming education: effects on learning performance, self-efficacy and processes. Australas. J. Educ. Technol. doi: 10.14742/ajet.9932

[ref17] Li Z. Wang C. Bonk C. J. (2026). Generative AI for teachers’ self-directed professional development: a mixed-methods study. TechTrends 70, 23–38. doi: 10.1007/s11528-025-01123-8

[ref18] Li H. Zhang Y. Chen M. Zhao T. Jou M. (2026). Creative personal identity in the age of generative AI: a social-cognitive pathway of AI literacy, self-efficacy, and mindset. Comput. Hum. Behav. 175:108838. doi: 10.1016/j.chb.2025.108838

[ref19] Mah D.-K. Groß N. (2024). Artificial intelligence in higher education: exploring faculty use, self-efficacy, distinct profiles, and professional development needs. Int. J. Educ. Technol. High. Educ. 21:58. doi: 10.1186/s41239-024-00490-1

[ref20] Mai L. T. T. Khue T. V. (2025). The impacts of students. Acceptance of ChatGPT on Their. 32, 1–14.

[ref21] Nazim M. Alzubi A. A. F. (2025). Empowering EFL teachers’ perceptions of generative AI-mediated self-professionalism. PLoS One 20:e0326735. doi: 10.1371/journal.pone.0326735, 40549814 PMC12185028

[ref22] Oran B. B. (2023). Correlation between artificial intelligence in education and teacher self-efficacy beliefs: a review. RumeliDE Dil ve Edebiyat Araştırmaları Dergisi 34, 1354–1365. doi: 10.29000/rumelide.1316378

[ref23] Pan Y. Li G. (2025). The effects of perceived teacher support and growth language mindset on learner well-being in AI-integrated environment: the mediating role of generative AI attitude. Front. Psychol. 16:1660462. doi: 10.3389/fpsyg.2025.1660462, 41035449 PMC12481513

[ref24] Shi L. (2025). Assessing teachers’ generative artificial intelligence competencies: instrument development and validation. Educ. Inf. Technol. 30, 23365–23384. doi: 10.1007/s10639-025-13684-5

[ref25] Tomczyk Ł. Majkut A. (2025). Integrating AI in education: an analysis of factors influencing the acceptance, concerns, attitudes, competencies and use of generative artificial intelligence among polish teachers. Hum. Behav. Emerg. Technol. 2025:5599169. doi: 10.1155/hbe2/5599169

[ref26] Xiang Y. Yang C. Jin Z. Zhao W. (2025). Factors influencing the adoption of generative artificial intelligence into classroom teaching by university teachers: an empirical study using SPSS PROCESS macros. PLoS One 20:e0324875. doi: 10.1371/journal.pone.0324875, 40833983 PMC12367140

