# Supplementary material for: Teachers’ competence, self-efficacy, and their attitudes toward generative AI in education: a correlational study
Source: Front Psychol. 2026 May 26;17:1756148. doi: 10.3389/fpsyg.2026.1756148 (PMC13247872; doi:10.3389/fpsyg.2026.1756148)
Supplement: Supplementary file 1 [file Supplementary_File_1.docx]

**Appendix: Survey Instruments**

All items were rated on a 5-point Likert scale: 1 = Strongly Disagree, 2 = Disagree, 3 = Neutral, 4 = Agree, 5 = Strongly Agree.

General Digital Competence (GDC) Scale (10 items)

1. I am proficient in using common digital productivity tools (e.g. word processors, spreadsheets, presentation software).
2. I can effectively find, evaluate, and use digital information from the internet for my teaching.
3. I am skilled at communicating and collaborating with colleagues and students using digital tools.
4. I am confident in my ability to use digital tools to create and share educational resources.
5. I know how to manage and organize digital files and data securely.
6. I can troubleshoot basic technical problems with classroom technology.
7. I am comfortable using a learning management system (LMS) to manage my courses.
8. I can integrate various digital media (e.g. video, audio, images) into my lessons.
9. I understand the basic principles of digital citizenship and can teach them to my students.
10. I stay updated with new digital technologies relevant to education.

AI-Specific Competence (AISC) Scale (8 items)

1. I have a basic understanding of what Generative AI is and how it works.
2. I understand the principles of effective prompt engineering to get desired outputs from a generative AI model.
3. I am aware of the potential for bias and inaccuracy in AI-generated content.
4. I know how to critically evaluate the quality and reliability of information provided by GenAI tools.
5. I am familiar with several different types of GenAI tools (e.g. text generators, image creators).
6. I understand the ethical issues related to using GenAI in education (e.g. plagiarism, data privacy).
7. I can identify appropriate and inappropriate uses of GenAI for student assignments.
8. I feel knowledgeable enough to guide students on the responsible use of GenAI.

Teacher Self-Efficacy for AI Integration (TSE-AI) Scale (10 items)

1. I am confident that I can learn the skills needed to use GenAI tools in my teaching.
2. I believe I can use GenAI to design engaging learning activities for my students.
3. I am confident in my ability to integrate GenAI into my existing curriculum.
4. I can find ways to use GenAI to support students with diverse learning needs.
5. I am confident I can manage a classroom where students are using GenAI tools.
6. I believe I can help students use GenAI to enhance their creativity and critical thinking.
7. I am confident in my ability to assess student work that has been created with the help of GenAI.
8. I can overcome the technical challenges that might arise when using GenAI in my classroom.
9. I am confident I can model the ethical and responsible use of GenAI for my students.
10. I believe I can adapt my teaching methods to effectively incorporate GenAI.

Attitudes Toward Generative AI in Education (ATGAI-E) Scale (12 items)

1. I believe GenAI will be a valuable tool for teachers in the future.
2. Generative AI has the potential to personalize learning for students.
3. Integrating GenAI into the classroom will enhance student engagement.
4. I am excited about the possibilities that GenAI brings to education.
5. GenAI can help reduce my workload by automating administrative tasks.
6. Learning to use GenAI is a worthwhile professional development goal for me.
7. I am concerned that the use of GenAI will hinder students' critical thinking skills. (R)
8. I worry that over-reliance on GenAI will lead to a decline in students' basic skills. (R)
9. I am concerned about the ethical implications of using GenAI with students (e.g. data privacy). (R)
10. I feel anxious about the rapid changes GenAI is bringing to the teaching profession. (R)
11. I am skeptical about the claimed benefits of GenAI in education. (R)
12. I believe the risks of using GenAI in schools outweigh the benefits. (R) *(R) indicates the item was reverse-coded for scoring.*
